# Alternative reproductive tactics increase effective population size and decrease inbreeding in wild Atlantic salmon

**DOI:** 10.1111/eva.12172

**Published:** 2014-06-18

**Authors:** Charles Perrier, Éric Normandeau, Mélanie Dionne, Antoine Richard, Louis Bernatchez

**Affiliations:** 1 Département de Biologie, Institut de Biologie Intégrative et des Systèmes (IBIS), Université LavalQuébec, Canada; 2 Direction de la faune aquatique, Ministère du Développement durable, de l'Environnement, de la Faune et des Parcs du QuébecQuébec, Canada

**Keywords:** effective number of breeders, Effective population size, genetic diversity, kinship, parentage analysis, relatedness, reproductive success, reproductive tactic.

## Abstract

While nonanadromous males (stream-resident and/or mature male parr) contribute to reproduction in anadromous salmonids, little is known about their impacts on key population genetic parameters. Here, we evaluated the contribution of Atlantic salmon mature male parr to the effective number of breeders (Nb) using both demographic (variance in reproductive success) and genetic (linkage disequilibrium) methods, the number of alleles, and the relatedness among breeders. We used a recently published pedigree reconstruction of a wild anadromous Atlantic salmon population in which 2548 fry born in 2010 were assigned parentage to 144 anadromous female and 101 anadromous females that returned to the river to spawn in 2009 and to 462 mature male parr. Demographic and genetic methods revealed that mature male parr increased population Nb by 1.79 and 1.85 times, respectively. Moreover, mature male parr boosted the number of alleles found among progenies. Finally, mature male parr were in average less related to anadromous females than were anadromous males, likely because of asynchronous sexual maturation between mature male parr and anadromous fish of a given cohort. By increasing Nb and allelic richness, and by decreasing inbreeding, the reproductive contribution of mature male parr has important evolutionary and conservation implications for declining Atlantic salmon populations.

## Introduction

The effective population size, Ne, is a central genetic parameter that determines the extent of inbreeding, genetic drift, and genetic diversity occurring within populations. As such, Ne also influences a population's evolutionary potential because it determines the efficiency of natural selection (Wright 1938; Charlesworth 2009). Inbreeding, the breeding between closely related individuals, is a major process influencing the fitness of populations due to the potential occurrence of inbreeding depression (Charlesworth and Charlesworth 1987). Moreover, the effectiveness of purifying selection on recessive deleterious alleles accumulated due to inbreeding (Crnokrak and Barrett 2002) can be reduced in populations experiencing small Ne (Crow and Kimura 1970). Hence, Ne and inbreeding are fundamental and interconnected population genetic parameters. Therefore, evolutionary and conservation studies of wild and captive populations increasingly consider Ne (Frankham 2005; Schwartz et al. 2007; Hare et al. 2011) and inbreeding (Hedrick and Kalinowski 2000; Frankham 2005; Edmands 2007).

Extensive variation in life-history traits, in particular in reproductive strategies, exists within populations of a species and can influence Ne, inbreeding, and ultimately the level of genetic variability that is maintained. Ne and the ratio of effective size over census size (Ne/Nc) are influenced by the sex ratio among breeders, the variance in individual reproductive success, the propensity for multiple mating, and the extent of overlapping generations (Wright 1938; Crow and Kimura 1970). For example, multiple mating can be negatively correlated to Ne if it is associated to an increase in variance in reproductive success (Ardren and Kapuscinski 2003). However, multiple mating can be positively correlated with Ne if it does not result in an increase in the variance in reproductive success but rather in an increase in the Ne of the limiting sex (Saura et al. 2008). Besides, multiple mating may dilute inbreeding and increase individual reproductive success (Stockley et al. 1993; Tregenza and Wedell 2002; Garant et al. 2005). Similarly, the decrease in synchronicity in sexual maturity among individuals from different sexes could reduce the probability of reproduction among siblings and thus limit inbreeding (Bukowski and Avilés 2002). Also, there are some evidences that inbreeding may also be actively avoided through active kin recognition and avoidance or indirectly through dispersal (Pusey and Wolf 1996; Kokko and Ots 2006). For example, while mature zebrafish male do not show preference for the odor of related or unrelated females, mature female prefer the odor of unrelated males (Gerlach and Lysiak 2006). Overall, the influence of alternative maturation and mating tactics on Ne and on inbreeding could be particularly critical within relatively small and isolated populations that are particularly sensitive to these genetic parameters and in which active kin avoidance may be particularly costly.

Salmonid fishes display extensive variation in breeding systems, exhibiting within and among species alternative reproductive strategies (Fleming 1998; Fleming and Reynolds 2004). In anadromous Atlantic salmon (*Salmo salar*, L.) populations, while all females achieve a migration at sea before homing to their natal river to spawn, variable proportions of males forego marine migration and reach sexual maturity at a small size in freshwater. These small males maturing in freshwater are usually named mature male parr. This reproductive strategy has strong environmental determinants but is also partially heritable (Prevost et al. 1992; Aubin-Horth and Dodson 2004; Piche et al. 2008; Paez et al. 2011; Dodson et al. 2013) and might have been by maintained through negative frequency-dependent selection (Hutchings and Myers 1994; Aubin-Horth et al. 2005). Using parental assignment, several studies have shown that mature male parr may fertilize relatively large proportions of eggs in the wild, ranging typically from 30% to 60% (Martinez et al. 2000; Saura et al. 2008; Grimardias et al. 2010; Richard et al. 2013). Mature male parr have low individual reproductive success compared to anadromous males but can be much more abundant and thus increase multiple mating (Martinez et al. 2000; Garant et al. 2003; Fleming and Reynolds 2004; Richard et al. 2013). Lastly, anadromous salmon and mature male parr from a single cohort will generally reproduce in different years, mature male parr being several years younger than mature anadromous males, thus increasing the overlap among generations. Mature male parr could also constitute a large reserve of genetic diversity as a result of their abundance. Lastly, the prevalence of inbreeding, which can be a major concern in salmonids (Wang et al. 2001; Houde et al. 2011), could be reduced by the contribution of mature male parr through the increase in Ne, the increase in multiple mating, and the increase in cohort overlap via asynchronous maturation with anadromous females.

Due to the impossibility of sampling all mature male parr in wild populations, estimating their influence on Ne and inbreeding is very difficult to do and has rarely been achieved. For the same reason, mature male parr are generally not included when estimating Nc, although they contribute to Ne. Yet, Ne has been measured by using pedigree reconstructions in either small experimental water channels (Jones and Hutchings 2002) or small wild populations (Saura et al. 2008). However, estimating the contribution of mature male parr to Ne in medium to large Atlantic salmon populations remains extremely challenging. This has only been performed by combining genetic (linkage disequilibrium) and demographic (number of anadromous breeders) estimates of Ne, to gain coarse estimates of the possible participation of mature male parr (Palstra et al. 2009; Johnstone et al. 2013). Nevertheless, pedigree reconstruction has sometimes been successfully used for relatively large populations in other species (e.g., *Oncorhynchus mykiss*, Ardren and Kapuscinski (2003), Araki et al. (2007) and *Salmo trutta*, Serbezov et al. (2012a)), demonstrating the feasibility of such a procedure. Moreover, in situations where all the potential anadromous Atlantic salmon breeders have been sampled, mature male parr presence and fertilization success can be deduced from pedigree reconstructions (Saura et al. 2008; Richard et al. 2013). Rather than Ne, which corresponds to the effective population size over a generation, the effective number of breeders for a given reproductive year, Nb, can be inferred from such pedigree reconstitution (Waples 2005; Palstra and Fraser 2012).

Here, using a recently published dataset (Richard et al. 2013), we document the contributions of mature male parr on the effective population size, genetic diversity and inbreeding in a medium size Atlantic salmon population. An exhaustive pedigree reconstruction of this North American Atlantic salmon population was achieved by assigning parentage, using 12 microsatellite markers, of 2548 fry collected in 2010 to 144 anadromous females and 101 anadromous males that returned to the river in 2009 and to 462 mature male parr for which genotypes were reconstructed using the Colony software (see Methods and Results). In this study, we first quantify the contributions of mature male parr to the effective number of breeders (Nb) using both demographic and genetic estimate of Nb, respectively, based on variance in reproductive success among breeders and on linkage disequilibrium among loci. Second, we empirically compare the sensitivity of both methods used to estimate Nb. Third, we quantify the bias in the estimate of Nb/Nc resulting from neglecting the spawning participation of mature male parr to breeding in the study population. Finally, we assess the contribution of mature male parr to the genetic diversity in terms of allelic richness among fry and we measure the impact of mature male parr on the relatedness among breeders, and consequently, on inbreeding within the population. We discuss the evolutionary and conservation implications of the contributions of mature male parr, and more widely of alternative maturation and mating tactics, to effective population size, inbreeding, and genetic diversity in wild salmonids populations and other species exhibiting extensive variation in life history.

## Methods

### Study site and sample collection

This study was conducted on the Escoumins River, Québec, Canada (River mouth coordinates: +48°20′ 48.20″N, −69°24′26.01″W). A dam built 1 km upstream of the river mouth, just upstream of tidal influence, and equipped with a trap allowed Richard et al. (2013) to sample all the anadromous genitors returning to the river to spawn. The average anadromous Atlantic salmon run size in this river was 312 (195–455) from 2004 to 2009. In 2009, all anadromous salmon entering the Escoumins River (*N* = 268) and passing the dam were measured and sampled (adipose fin tissue). In August 2010, 2577 fry (young of the year, 0+) were randomly sampled by electrofishing at 94 sites. More methodological details pertaining to study site and sampling can be found in the study described by Richard et al. (2013).

### Genetic analyses and parentage analysis

The genetic and parentage analyses are also detailed in the study of Richard et al. (2013). Briefly, DNA was extracted from adipose fin tissue and from fry caudal fin tissue using a salt-based method (Aljanabi and Martinez 1997). Microsatellite polymorphism was analyzed at 12 loci. All adult individuals were genotyped twice (from PCR to genotyping) to prevent genotyping errors. Potential presence of null alleles, large allele dropout, and stutter peaks were estimated using MICROCHECKER 2.2.3 (Van Oosterhout et al. 2004).

**Figure 1:**
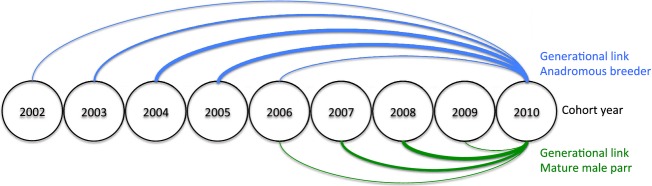
Schematized life cycle of the Atlantic salmon population in the Escoumins River. This life cycle illustrates the potential generational links between the fry sampled in 2010 and the anadromous or mature male parr breeders from the most probable anterior cohorts.

Figure1 illustrates a typical life cycle of the Atlantic salmon population inhabiting the Escoumins River, showing the potential cohorts of anadromous and mature male parr breeders that may have contributed to the fry sampled in 2010. CERVUS and PASOS (Duchesne et al. 2005) were first used to find the most likely mother–offspring and father–offspring pairs. A total of 1247 paternities and 2395 maternities were identified. Then, the full likelihood approach implemented in COLONY (Jones and Wang 2010) was used to allocate a mother or a father to the 153 and 1301 remaining fry, respectively. Probability of identity and paternity exclusion probability combined across all loci was 5 × 10^−6^ for the first parent, allowing high-quality assignments (see Richard et al. 2013 for further details on assignment reliability). Although mature male parr were not sampled, COLONY was used to infer their genotypes from the pedigree analysis using a 0.60 probability that a father was present among the anadromous fish. To determine the effect of the number of offspring sampled on the potential for detection of anadromous parents and of mature male parr fathers, we subsampled from 50 to 2500 offspring by steps of 50, with each step being subsampled 1000 times. The number of parents identified should reach a plateau if enough offspring were sampled.

Based on this parental assignment analysis, Richard et al. (2013) reported that of the 268 anadromous Atlantic salmon that entered the river during the summer of 2009 (Table S1), 245 (101 males and 144 females) had at least one fry assigned to them by our parentage analysis. In addition, a total of 462 mature male parr were identified as breeders. Of the 2548 fry that were assigned parentage, 1115 (44%) were assigned to mature male parr and 1433 (56%) to anadromous males (Table S1). Mature male parr therefore increased the number of breeding males by 5.63 times and the total number of breeders by 2.97 times. It is noteworthy that the progeny of only six of the 144 females were assigned to only mature male parr.

### Demographic estimates of the effect of mature male parr on the effective number of breeders (Nb)

Based on the parentage assignments, we estimated the mean (*k*) and variance (*Vk*) of reproductive success among parents of a same sex. Because not all the fry were sampled, we adjusted the observed index of variability (*Vk*[obs]/*k*[obs]) to obtain an adjusted value *Vk*[adj]/*k*[adj] using the eqn (1), for each sex separately:
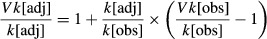
1

Where *Vk*[adj] is the adjusted variance in reproductive success (Crow and Morton 1955; Waples 2002), following the methods of Araki et al. (2007). Assuming a stable population size, *Vk*[adj] was adjusted for *k* = 2.

We then estimated the Nb for each sex following the eqn (2).


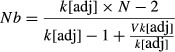
2

The overall effective number of breeders was then estimated using eqn (3) (Crow and Kimura 1970).


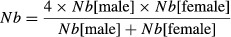
3

To investigate further the contribution of mature male parr individuals on Nb, we estimated Nb (referred as Demo(1)) for two different datasets corresponding to parents-fry assignments implicating (i) anadromous breeders and (ii) anadromous and mature male parr breeders.

Mature male parr and anadromous males are likely to compete for egg fertilization (Myers and Hutchings 1987). Therefore, estimates of Nb based on only anadromous males could be biased due to an underestimation of females’ reproductive success since their eggs has been partly fertilized by mature male parr. We thus performed a second demographic estimate of Nb, referred as Demo(2), using the first aforementioned scenario Demo(1) but simulating the possibility that fry fathered by mature male parr could have been fathered by anadromous males. To do so, we replaced the mature male parr by anadromous males in the parentage results, proportionally to the reproductive success of anadromous males with each female. To consider that some males may have equal reproductive success, this replacement procedure was run 1000 times *in silico*. We then estimated Nb for this new parentage file using the aforementioned equations.

### Genetic estimates of the effect of mature male parr on the effective number of breeders (Nb)

We used the LDNe program (Waples and Do 2008) to estimate Nb (referred as Nb LDNe) and the contribution of anadromous males and mature male parr individuals to Nb and. We excluded alleles with frequencies below 0.01 and estimated 95% confidence intervals using the parametric test implemented in LDNe.

### Effect of mature male parr on Nb/Nc

We estimated the Nb/Nc ratios by dividing Nb by either (i) the number of anadromous individuals counted at the dam or (ii) the number of breeders identified by parental assignments, including mature male parr.

### Estimates of the sensitivity of Nb estimates to the number of fry sampled

To investigate the influence of the number of fry sampled on the demographic estimate of Nb, we estimated Nb (Nb Demo(1)) for the two above-mentioned datasets, subsampling from 100 to 2500 progeny by steps of 100 progeny, with 1000 subsamples for each step and without replacing individuals. To investigate the influence of the number of fry sampled on the genetic estimate of Nb (Nb LDNe), we ran the software on random subsets of 100 to 2500 fry (with increments of 100 progeny) resampled 100 times without replacing individuals.

### Estimate of the effect of mature male parr on genetic diversity in the progeny

We hypothesized that a greater contribution of mature male parr to allelic richness could be mediated by their large number associated with their small individual reproductive success compared to anadromous males. We therefore compared the total number of alleles found among progeny assigned to (i) anadromous pairs, (ii) anadromous female × mature male parr and (iii) both types of pairs, for an increasing numbers of progeny sampled. We subsampled from 50 to 2500 fry, by steps of 50, 100 times for each step, and estimated the total number of alleles found among fry assigned to the three aforementioned groups of parents. To graphically represent the differences in the number of alleles among these three groups, we represented a Loess regression of the median value of the total number of alleles found for each number of fry sampled considered, as well as the 5–95% interval distribution of the data.

### Estimate of the effect of mature male parr on inbreeding

We investigated the effect of mature male parr on inbreeding by comparing the Loiselle et al. (1995) kinship coefficient estimated for the three following groups of breeders’ pairs: (i) anadromous breeders, (ii) anadromous female × mature male parr pairs and (iii) both type of pairs. We computed the Loiselle coefficient using the software GENODIVE (Meirmans and Van Tienderen 2004). First, we compared average kinship coefficients among breeders within each pair for (i) anadromous pairs, (ii) anadromous female × mature male parr and (iii) both type of pairs. Each pair's kinship coefficient had the same weight. This first comparison allowed investigating the effect of the mating strategy on the relatedness among breeders. Second, for these three same groups of pairs, we compared kinship coefficients weighted by the relative reproductive success of each pairs. Weighting each pairs’ kinship coefficient was achieved to assess the potential outcomes of the mating strategies on the next cohort. Second, we compared the Lynch (Lynch and Ritland 1999) inbreeding coefficients in fry produced by the three same groups of breeders’ pairs as defined above. We computed Lynch coefficients using ML-RELATE (Kalinowski et al. 2006). We tested the significance in mean differences using *T*-tests implemented in R.

## Results

### Estimates of the impact of mature male parr on the effective number of breeders (Nb) and on Nb/Nc

**Figure 2:**
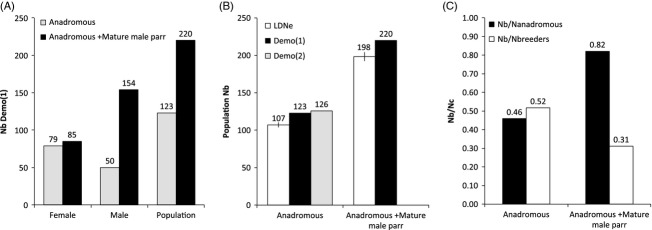
(A) Demographic estimates of effective number of breeders (Nb Demo(1)) for males, females, and the entire population, considering either only anadromous salmon or both anadromous fish and mature male parr. (B) Genetic (LDNe) and demographic estimates (Demo(1) and Demo(2)) of effective number of breeders considering either only anadromous salmon or both anadromous fish and mature male parr. There is no Demo(2) estimate when considering anadromous fish and mature male parr since all males were already considered in this estimation. Confidence intervals are drawn for LDNe estimates. (C) Ratio Nb/Nc for either only anadromous salmon or both anadromous fish and mature male parr. Nc corresponds either to the number of anadromous fish entering the river or to the number of breeders identified by parentage analyses.

Demographic estimates of male Nb were 50 without and 154 with mature male parr (Fig.2A, Table S1). Female Nb was 79 without and 85 with mature male parr. Population Nb was 123 without and 220 with mature male parr. Therefore, male Nb, female Nb and population Nb were 3.07, 1.07, and 1.79 times larger when mature male parr were included. Using the replacement method Demo(2), the population Nb increased by only 3%, (Fig.2B, Table S1), suggesting a modest bias in estimates of Nb originating from potential male competition.

Using LDNe to estimate Nb from the genetic data, we obtained values of 107 without and 198 with mature male parr, with confidence intervals of 5.6 and 11.7 (Table S1, Fig.2B). The width of the confidence intervals varied from 5.6 to 11.7, with an average of 8.7. The Nb values estimated using LDNe were thus very similar to the one estimated with the demographic method Demo(1), though lower by 10% in average (Nb = 107 and 123 without mature male parr and 198 and 220 with mature male parr for genetic and demographic methods, respectively; Table S1, Fig.2B).

The Nb/Nc ratios were 0.46 without and 0.82 with precocious parr to estimate Nb, respectively when we used Demo(1) to estimate Nb and included only anadromous fish in Nc (Table S1, Fig.2C). Thus, including mature male parr into estimates of Nb but not Nc increased the Nb/Nc ratio. In contrast, when all the effective breeders (anadromous and mature male parr that had progeny assigned) were considered within Nc, the Nb/Nc ratios were 0.52 and 0.31, without and with precocious parr, respectively. Thus, including mature male parr to estimate both Nb and Nc reduced the Nb/Nc ratio through a more substantial increase in Nc relative to Nb.

### Influence of the number of analyzed individuals on Nb estimates

**Figure 3:**
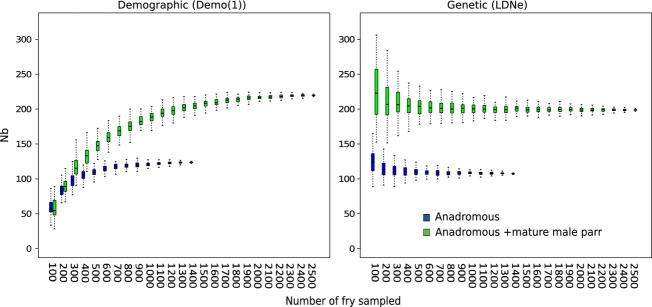
Boxplot of population's Nb estimates obtained from demographic (Demo(1)) and genetic (LDNe) methods for either only anadromous salmon or both anadromous fish and mature male parr with 100–2500 (increments of 100 progeny) progeny subsampled 1000 and 100 times for Demo(1) and LDNe, respectively.

We compared demographic (Nb Demo(1)) and genetic estimates (Nb LDNe) of Nb for various numbers of progeny (Fig.3). As expected, the variance in Nb estimates decreased when an increasing number of progeny were resampled for both methods. The median values of the demographic estimates increased from 100 to 1500 juveniles sampled and then reached a plateau (Fig.3A). For instance, median values of Nb were 55, 148, and 208 for 100, 500, and 1500 progeny subsampled, respectively, (anadromous individuals and mature male parr included). In contrast, the median values for the genetic estimates varied only slightly with the number of progeny (Fig.3B). Median values of Nb were 223, 204, and 199 for 100, 500, and 1500 progeny subsampled, respectively, (anadromous individuals and mature male parr included). Similar trends were observed for datasets including only anadromous salmon or both anadromous fish and mature male parr.

### Estimate of the effect of mature male parr on genetic diversity among progeny

**Figure 4:**
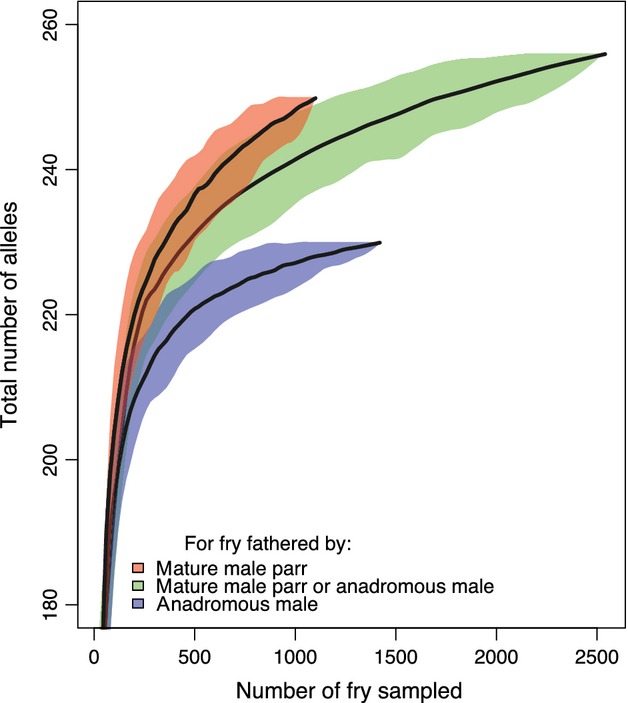
Loess regression of the median value of the number of alleles found for 50–2500, fry for fathers corresponding to (i) mature male parr, (ii) anadromous male or mature male parr and (iii) anadromous male. Each estimate was bootstrapped 100 times. Five to 95% interval distribution of the data were given around the median value.

Figure4 illustrates a higher number of alleles among fry produced by pairs of anadromous females and mature male parr than for fry produced by pairs of anadromous fish. It also shows a lower number of alleles among fry produced by anadromous pairs than among fry produced by both anadromous pairs and that produced by anadromous females and mature male parr. For instance, sampling 1000 progeny 100 times, the average number of allele were 227 (SD = 1.44), 241 (SD = 2.95), and 248 (SD = 1.39) when considering progeny assigned to anadromous pairs, anadromous female and mature male parr, and both type of pairs, respectively. This figure also shows how the difference in the number of alleles among groups increases with the number of fry sampled. Overall, the total number of alleles was 230 and 256 without and with mature males parr.

### Estimates of the effect of mature male parr on inbreeding

**Figure 5:**
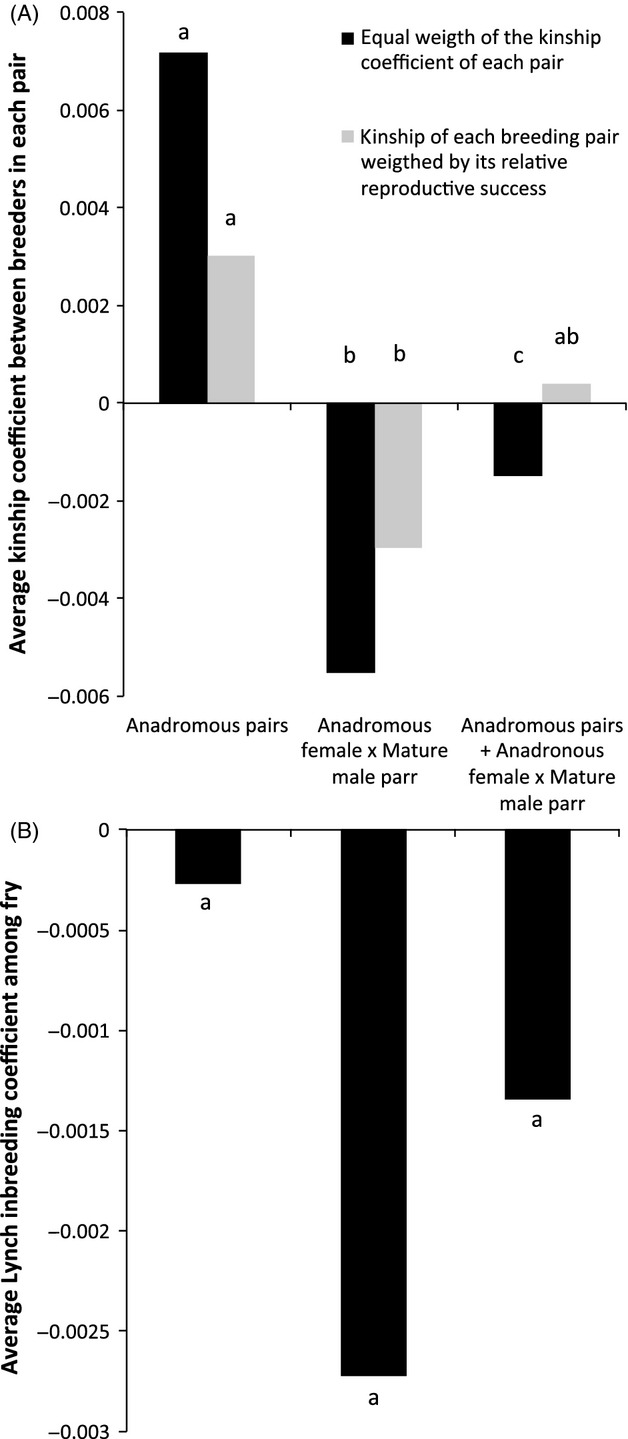
(A) Average Loiselle kinship coefficient among breeders within each pair for (i) anadromous pairs, (ii) anadromous female × mature male parr pairs and (iii) both type of pairs. Kinship coefficients were given with and without weighting each pair's kinship value by the relative reproductive success of the given pair. (B) Average Lynch inbreeding coefficient among fry assigned to (i) anadromous pairs, (ii) anadromous female × mature male parr pairs and iii) both type of pairs. Letters indicates statistical differences when performing *t*-tests.

The Loiselle kinship coefficient was significantly higher among pairs of anadromous breeders (0.0072) than among pairs of anadromous female and mature male parr (−0.0055) (*T*-test, *t* = 3.87, *P *<* *0.001) (Fig.5A). The kinship coefficient was also higher on average among pairs of anadromous breeders compared with all type of pairs taken together (−0.0015) (*T*-test, *t* = 2.80, *P *<* *0.01). When adjusted for the relative reproductive success of each pair, kinship coefficient was on average higher among pairs of anadromous breeders (0.0030, Fig.4A) than among pairs of anadromous female and mature male parr (−0.0030) (*T*-test, *t* = 2.65, *P *<* *0.01). However, no significant difference was found in the average kinship coefficient among pairs of anadromous breeders compared to all type of pairs taken together (0.0004) (*T*-test, *t* = 1.49, *P *=* *0.14). Estimates of Lynch's inbreeding coefficients were not different between fry produced by pairs of mature male parr and anadromous females compared to anadromous pairs (*T*-test, *t* = 0.85, *P *=* *0.39) (Fig.5B).

## Discussion

Our study yields five important insights into the influence of nonanadromous males on key population genetic features in Atlantic salmon. First, it empirically illustrates the substantial increase in precision obtained for both demographic and linkage disequilibrium estimates of Nb with the number of samples used. Second, we found that mature male parr increased male Nb and population Nb by 3.07 and 1.79 times, respectively. Third, Nb/Nc was overestimated by at least 2.6 times when mature male parr were overlooked. Fourth, mature male parr significantly increased the number of alleles found among the progeny. Finally, mature male parr decreased the average relatedness among breeders. These results have consequential evolutionary and conservation implications.

### Comparisons between demographic and genetic estimates of the effective number of breeders (Nb)

While demographic and genetic methods used to estimate Nb technically differ, the Nb estimates calculated with these were quite similar, illustrating the strength of these approaches when applied to large datasets. Our results are in line with the study of Serbezov et al. (2012b) showing modest differences only between genetic (Nb = 53) and demographic (Nb = 40) Nb estimates in a small *Salmo trutta* population. Hoehn et al. (2012) also found comparable estimates of Nb using demographic and genetic estimates in a gecko species. Such comparisons of demographic and genetic estimates, however, remain relatively rare because the demographic method requires collecting and genotyping all or most of the potential parents in a population or otherwise having direct observations on the number and sex of parents and their reproductive success.

Although both genetic and demographic methods yielded similar estimates of Nb when applied to the entire dataset, the demographic method was very sensitive to the number of progeny used to estimate reproductive success, contrasting with the robustness of the genetic method. Indeed, our resampling procedure illustrated that the Nb estimates reached a plateau at 700 and 2000 progeny for anadromous males and mature male parr, respectively. Under these thresholds, Nb estimates were found increasingly underestimated with the decrease in the number of progeny considered. This can be explained by the need of large number of progeny to estimate the variance in reproductive success among breeders. In particular, given the potentially large number of mature male parr contributing to reproduction and because of their small individual reproductive success, each additional progeny sampled increased the chance to detect the participation of a mature male parr and thus to enrich the measure of their contribution to Nb. Hence, the number of progeny which were assigned parentage in Serbezov et al. (2012a) and in this study were, respectively, 3.0 and 3.6 times the total number of breeders identified (9.5 times the number of anadromous salmon entering the river in the study). Accordingly, such demographic estimate of Nb is limited to relatively small populations and/or exhaustive sampling. This is particularly true when an alternative mating strategy implicates a large number of breeders having small individual contributions.

Even though exhaustive parental assignment were here required to link reproductive tactics (anadromous versus precocious male parr) to Nb, genetic estimates based on linkage disequilibrium among loci (Hill 1981; Waples and Do 2008) were much more robust than the demographic ones to the number of progeny subsampled. Indeed, using linkage disequilibrium signals found in 200 progeny (0.75 times the number of anadromous breeders, 0.28 times the total number of breeders) was sufficient to estimate Nb quite precisely. In addition, confidence intervals for the Nb estimated for progeny fathered by anadromous fish and mature male parr did not overlapped from 200 progeny subsampled. This precision obtained with the linkage disequilibrium method is in line with several recent studies reporting that this approach can be applied to small representative subset of individuals from small panmictic populations (Tallmon et al. 2010; Waples 2010; Waples and Do 2010; Antao et al. 2011; Whiteley et al. 2012). However, a relatively large variance in Nb estimates and a small overestimation were observed for a low value of 100. This variance could have been partly caused by unequal representation of families (Whiteley et al. 2012). Indeed, we did not take the geographic origin of progenies in consideration in our bootstrapping procedure to simplify it. Overall, this empirical comparison of demographic and linkage disequilibrium methods therefore adds to previous recommendations regarding sampling strategy and genotyping effort for precise estimate of Nb (Tallmon et al. 2010; Whiteley et al. 2012) but also for comparing the effect of different life history tactics on Nb.

### Contribution of mature male parr to the effective number of breeders (Nb)

The participation of mature male parr in breeding resulted in a 3.07-fold increase in male Nb and a 1.79-fold increase in population Nb. These increases in Nb are lower than those reported by Saura et al. (2008) for Atlantic salmon, who found a 10-fold increase in male Nb and a two- to threefold increase in population Nb when mature male parr were included in Nb estimates. The differences in Nb increases between both studies may be principally due to the higher proportion of fry assigned to parr in Saura et al. (2008) compared to this study (60% and 44%, respectively). Nevertheless, it is also possible that differences between studies may be due to differences in census size (Nc anadromous fish ∼ 41, compared to 268 here) and a much smaller number of progeny that could be assigned parentage (∼90, compared with 2548 here; corresponding to 4.3 times less progeny per anadromous fish). Our results were more consistent with those of Johnstone et al. (2013) showing that mature male parr increased male Nb by a factor of 3.2. Important contributions of mature male parr to Nb have also been documented experimentally (Jones and Hutchings 2002), but values cannot be directly compared because the numbers of mature male parr introduced in these experiments were relatively small compared with numbers observed in the wild (Weir et al. 2010; Richard et al. 2013). Even if our assignment analysis was extensive compared with previous studies on Atlantic salmon (Jones and Hutchings 2001; Saura et al. 2008), it is unlikely that we identified all mature male parr present in the river.

Similarly to Saura et al. (2008), we found that the increase in male Nb due to the contributions from mature male parr had little impact on female Nb. This can first be explained by the usual involvement of at least one anadromous male mating with anadromous female (Fleming and Reynolds 2004; Richard et al. 2013). Accordingly, we found only six females, which mated with only mature male parr. Second, females which mated with many mature male parr increased their average reproductive success but also increased the variance in their reproductive success, leading to only a small increase in female Nb. Overall, although they had a low individual reproductive success (in absolute terms) compared with their anadromous counterparts, mature male parr increased population Nb by increasing census size and taking part in multiple matings.

Focusing on a single cohort allowed an efficient estimate of the contribution of mature male parr to the number of breeders, but their contribution to Ne may differ for several reasons. First, their contribution to Ne could be influenced by temporal fluctuations in the proportion of parr maturing that notably depends on environmental conditions (Herbinger and Friars 1992; Aubin-Horth and Dodson 2004; Piou and Prevost 2013). In addition, temporal fluctuations in the relative numbers of breeders from each category may affect operative sex ratio and thus Nb and Ne. Second, Atlantic salmon is not a semelparous species and exhibits some generational overlap. However, iteroparity rates remain generally low, varying between 0% and 10% (Fleming 1996). The influence of mature male parr on Ne should thus be similar to that on Nb since Ne and Nb * *Generation length* are very similar in the case of semelparous species (Waples 1990) and for iteroparous species with low iteroparity rates (Hare et al. 2011). Nevertheless, even though the mortality of mature male parr can be increased by up to 56% compared with same age nonmaturing parr (Myers 1984), a proportion of them are able to achieve a migration at sea (Myers 1984; Hutchings and Myers 1987) and can thus reproduce both as parr and several years later as anadromous fish. Consequently, the contribution of mature male parr to Ne could be slightly lower than to Nb, as shown in brown trout (Serbezov et al. 2012a). Moreover, mature male parr are younger than the anadromous females they breed with, thus potentially contributing to an increased genetic diversity and potentially Ne through mating between cohorts (Juanes et al. 2006). Future studies should aim at evaluating the contribution of mature male parr to effective population size over one or multiple generations, using lifetime rather than seasonal reproductive success, obtained by parental analyses over temporal monitoring of wild populations.

### Mature male parr and the ratio of effective number of breeders to census size (Nb/Nc)

While variable proportions of mature male parr contribute to Nb, neither their abundance nor their reproductive successes relative to anadromous fish are usually quantified, resulting in underestimates of Nc and thus in overestimates of Nb/Nc. In the present study, the Nb/Nc ratio estimated using the demographic method changed from 0.82 to 0.31 when mature male parr were included in Nc, confirming that estimates of Nb/Nc in Atlantic salmon are biased when the breeding contribution of mature male parr is not taken into account (Palstra et al. 2009; Johnstone et al. 2013; Moore and Fraser 2013). On one hand, such biased Nb/Nc ratio may limit the comprehension of the mechanisms by which Nb differs from Nc. On the other hand, as one of the central aims of estimating Nb using molecular markers is to extrapolate Nc for conservation issues (Luikart et al. 2010), one could assume that such constant overestimation of Nb/Nc might not bias Nc estimates. However, this overestimation of Nb/Nc may be far from constant in time and space. Indeed, mature male parr have variable abundance and reproductive success relative to anadromous fish depending on the breeding population and latitude but also through time in a same river (Valiente et al. 2005; Dodson et al. 2013). It therefore appears critical to estimate to which extent such alternative mating strategies usually not considered in Nc may bias Nb/Nc and Ne/Nc estimates in various populations and over time.

### Influence of mature male parr on genetic diversity and inbreeding

To our knowledge, this is the first study illustrating that mature male parr lead to an increase in the number of alleles observed in progeny (by 11%). Moreover, the number of alleles found in the progeny of anadromous pairs was lower than that observed among the fry produced by pairs including anadromous females and mature male parr. The larger number of mature male parr and their lower individual reproductive success compared with anadromous males can explain this result. This diversity could have also been slightly enhanced by asynchronous maturation between mature male parr and anadromous males.

Similarly, this study is also the first to quantify the contribution of mature male parr to reducing relatedness among parents and inbreeding in fry in Atlantic salmon. While inbreeding was not significantly different between progeny fathered by mature male parr and anadromous males, relatedness was in average smaller between mature male parr and anadromous female than between anadromous partners. We thus propose that alternative sexual maturation strategies may result in reduced relatedness among breeders as well as long-term inbreeding depression at the population level. In salmonids, dispersal and active kin avoidance could play a role in inbreeding reduction. However, homing can be extremely accurate and gene flow low even between relatively close subpopulations of a single river-system (Dionne et al. 2008; Vaha et al. 2008). Similarly, while active mate choice based on MHC-related genes diversity has been suggested in salmonids (Landry et al. 2001), active kin avoidance among anadromous breeders has never been demonstrated. Two main mechanisms diminishing inbreeding in Atlantic salmon without implying an active, and selected for, kin avoidance can thus be proposed: multiple mating and overlap among generations. In the absence of mature male parr and in semi-natural conditions, Garant et al. (2005) suggested that multiple mating increased individual reproductive success and the proportion of outbred progeny in female Atlantic salmon. Although generation overlap can be provided by differences in age structure in anadromous fish, we propose that mating between anadromous females and asynchronously maturing male parr of an earlier cohort can be an effective way to decrease inbreeding. While mature male parr are most commonly 1+ or 2+ old (Dalley et al. 1983; Fig.1), anadromous breeders are predominantly 4+, 5+, or 6+ (Palstra and Dionne 2011; Fig.1), respectively. Therefore, while it is possible for anadromous breeders to breed with half-sibs or full sibs, it is virtually impossible for an anadromous female to mate with a mature male parr of same sex. Given the negative relationship between individuals’ inbreeding and fitness previously documented in salmonids (Wang et al. 2001; Houde et al. 2011; Naish et al. 2013), such a strategy minimizing inbreeding without imposing a reproductive cost through kin avoidance might increase individual fitness, especially in the case of small populations where inbreeding is expected to rise. While the decrease of inbreeding associated with asynchronous maturation has been shown in subsocial spiders (Bukowski and Avilés 2002), this phenomenon has rarely been proposed in other animal taxa. Hence, future studies could further investigate the effect of asynchronous maturation in salmonids by linking parent age differences to their relatedness, their reproductive success and inbreeding in fry.

### Implications for maintaining evolutionary potential and long-term persistence of populations

The increase in Nb and genetic diversity and the decrease in inbreeding caused by mature male parr have important implications for the management and long-term persistence of wild Atlantic salmon populations. From a demographic view, mature male parr constitute a male reservoir in freshwater that could compensate for fluctuations in the number of anadromous males returning from long spawning migrations. Compensation among reproductive forms has been shown to be responsible for limiting Ne fluctuations in other species (e.g., steelhead trout, (Ardren and Kapuscinski 2003; Araki et al. 2007). When only relatively few anadromous male breeders survive until breeding, participation of mature male parr to reproduction may limit the decrease in Ne, the loss of genetic diversity, and contribute to reduce relatedness among fry. This may be especially important in the context of recreational fishing of Atlantic salmon in which generally only one-sea-winter fish, that are mainly males, can be kept. Moreover, the potential common overestimation of Nb/Nc should be carefully considered when such a ratio is taken into account for management purposes. As suggested by (de Mestral et al. 2012), it is important to consider mature male parr when estimating the number of breeders, especially when the number of anadromous fish, especially males, is low.

From an evolutionary perspective, this study confirms that mature male parr could contribute to the maintenance of evolutionary potential in wild salmonid populations. Assuming similar contributions of mature male parr to Nb and Ne, mature male parr may contribute to a global increase in Ne over the long term. A higher Ne may in turn insure greater and more stable population genetic diversity (Johnstone et al. 2013). In fact, the contribution of a large number of mature male parr to reproduction breeding effectively increased neutral genetic diversity at the loci considered in this study. Hence, Ne influences the rate of change in the genetic composition of a population due to drift and the effectiveness of selection relative to drift (Charlesworth 2009; Olson-Manning et al. 2012). A higher effective population size can also prevent the loss of standing variation via the fixation of neutral or nearly neutral alleles. These alleles may later be selected for if the local environment changes or during dispersal into marginal or new environments (Kawecki 2000; Lenormand 2002; Oakley 2013). Similarly, the reduction of inbreeding caused by the contribution of mature male parr could also reduce the risks of decrease in fitness associated with inbreeding depression (Wang et al. 2001; Naish et al. 2013). Eventually, the impact of mature male parr on the effective population size, genetic diversity, and inbreeding may contribute to limit the risk of extinction in small Atlantic salmon populations (Frankham 2005). More globally, this study illustrates how demographic and genetic contributions of alternative reproductive strategies should be carefully considered from both evolutionary and conservation perspectives.

## Data archiving statement

Data for this study is available from the Dryad Digital Repository: http://dx.doi.org/10.5061/dryad.20cf2.

## Supporting Information

Table S1 Demographic and genetic estimates of the breeding contribution of anadromous salmon and mature male parr to the effective number of breeders (Nb) and the Nb/Nc ratio in the Escoumins River in 2009.
