# Supplementary material for: Alternative reproductive tactics increase effective population size and decrease inbreeding in wild Atlantic salmon
Source: Evol Appl. 2014 Jun 18;7(9):1094–106. doi: 10.1111/eva.12172 (PMC4231598; doi:10.1111/eva.12172)
Supplement: Table S1 — Demographic and genetic estimates of the breeding contribution of anadromous salmon and mature male parr to the effective number of breeders (Nb) and the Nb/Nc ratio in the Escoumins River in 2009. [file eva0007-1094-sd1.docx]

Table S1. Demographic and genetic estimates of the breeding contribution of anadromous salmon and mature male parr to the effective number of breeders (Nb) and the Nb/Nc ratio in the Escoumins River in 2009. This table also details the number of individuals (N or Nc), reproductive success (k) and variability indexes (vk/k) used to estimate Nb and Nb/Nc by the demographic methods. See methods for the detail of the Nb estimates.

|  | Anadromous | | Anadromous  +Mature male parr | |
| --- | --- | --- | --- | --- |
|  | male | female | male | female |
| *N individuals – demographic method (1)* | | |  |  |
| N anadromous fish | 268 | | 268 | |
| N anadromous breeders | 101 | 138 | 101 | 144 |
| N mature parrr breeders | 0 | na | 462 | na |
| N breeders | 239 | | 707 | |
| N off assigned | 1408 | | 2548 | |
| *Reproductive success – demographic method (1)* | | |  |  |
| k obs | 14,19 | 10,31 | 4,53 | 17,69 |
| vk obs | 210,03 | 87,96 | 58,61 | 229,5 |
| [vk/k] obs | 14,80 | 8,53 | 12,95 | 12,97 |
| [vk/k] adj for k=2 | 2,95 | 2,46 | 6,28 | 2,35 |
| *Nb sex - demographic method (1)* | |  |  |  |
| Nb demo sex | 50 | 79 | 154 | 85 |
| *Nb - demographic methods* | |  |  |  |
| Nb demo (1) | 123 | | 220 | |
| Nb demo (2) | 126 | | - | |
| *Nb / N - demographic methods* | |  |  |  |
| Nb demo / N breeders (1) | 0,52 | | 0,31 | |
| Nb demo / N anadromous (1) | 0,46 | | 0,82 | |
| Nb demo / N breeders (2) | 0,53 | | - | |
| Nb demo / N anadromous (2) | 0,47 | | - | |
| *Nb - genetic method* | |  |  |  |
| Nb LDNe | 107 [103.9-110.5] | | 198 [192.4-204.1] | |
| *Nb / N - genetic method* |  | |  | |
| Nb LDNe / N breeders | 0,45 | | 0,28 | |
| Nb LDNe / N anadromous | 0,4 | | 0,74 | |
